# Prespacer processing and specific integration in a Type I-A CRISPR system

**DOI:** 10.1093/nar/gkx1232

**Published:** 2017-12-08

**Authors:** Clare Rollie, Shirley Graham, Christophe Rouillon, Malcolm F White

**Affiliations:** Biomedical Sciences Research Complex, School of Biology, University of St Andrews, North Haugh, St Andrews, Fife KY16 9ST, UK

## Abstract

The CRISPR–Cas system for prokaryotic adaptive immunity provides RNA-mediated protection from viruses and mobile genetic elements. Adaptation is dependent on the Cas1 and Cas2 proteins along with varying accessory proteins. Here we analyse the process in *Sulfolobus solfataricus*, showing that while Cas1 and Cas2 catalyze spacer integration *in vitro*, host factors are required for specificity. Specific integration also requires at least 400 bp of the leader sequence, and is dependent on the presence of hydrolysable ATP, suggestive of an active process that may involve DNA remodelling. Specific spacer integration is associated with processing of prespacer 3′ ends in a PAM-dependent manner. This is reflected in PAM-dependent processing of prespacer 3′ ends *in vitro* in the presence of cell lysate or the Cas4 nuclease, in a reaction consistent with PAM-directed binding and protection of prespacer DNA. These results highlight the diverse interplay between CRISPR–Cas elements and host proteins across CRISPR types.

## INTRODUCTION

**Figure 1.**
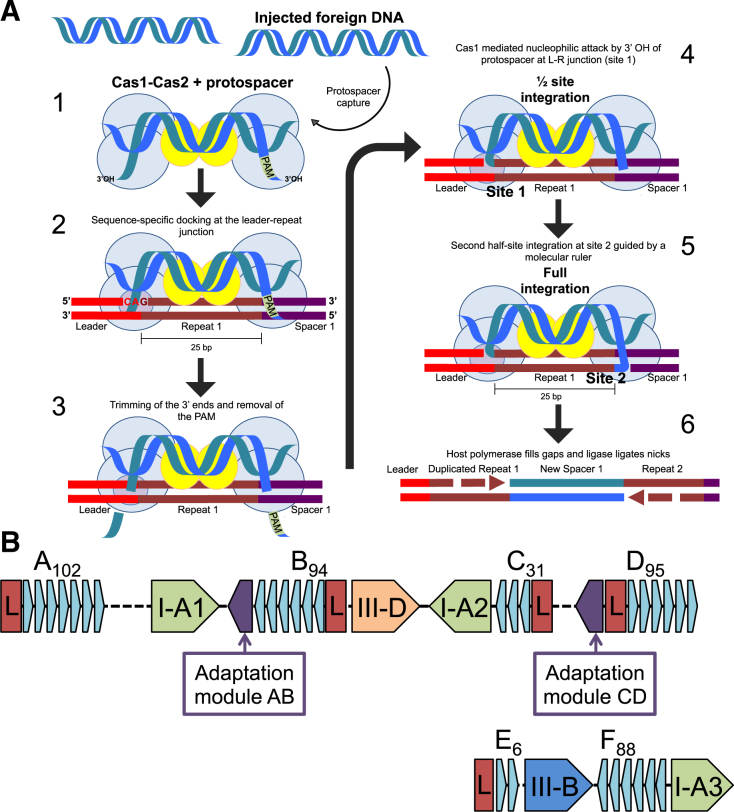
Adaptation in *S. solfataricus*. (**A**) Model for the integration of a new spacer by Cas1–Cas2 during adaptation (based on (1)). 1. A short segment of DNA containing a PAM sequence is captured and bound by a complex of Cas1 and Cas2 proteins. The ends of the captured prespacer may be splayed and trimmed by nucleases. 2. The prespacer-bound adaptation complex docks sequence-specifically at the leader-repeat junction of the host CRISPR array. The PAM may provide a polarity to the complex, as the PAM-proximal end of the prespacer must be integrated at the leader-distal end of repeat 1 (site 2) to allow transcription of crRNA in the correct orientation for interference. 3. During integration of the prespacer the 3′ ends may be trimmed. 4. A transesterification reaction mediated by Cas1 joins one 3′ hydroxyl of the incoming spacer to the leader-proximal 5′ end of 1st repeat (site 1). 5. A second transesterification joins the other end of the prespacer to the 5′ repeat end at site 2. 6. Gap filling and ligation. (**B**) Representation of the CRISPR–Cas system of *S. solfataricus*. The Cas1_AB_ and Cas2_AB_ proteins are encoded by the *sso1405* and *sso1404* genes associated with CRISPR loci A and B. The *sso1450* and *sso1450a* genes encoding Cas1_CD_ and Cas2_CD_ are associated with CRISPR loci C and D. The number of spacers contained in the CRISPR arrays is indicated in subscript after the array name. Leader regions are shown in red and indicated by the letter ‘L’. There are three type I-A, one type III-B and one type III-D effector modules.

CRISPR–Cas systems are present in around half of bacterial and 90% of archaeal genomes sequenced to date and form an adaptive immune system important in defence against invasion by foreign nucleic acids. Key to CRISPR–Cas immunity is the ability to adapt to new threats by incorporating short segments of foreign DNA, called spacers, into the CRISPR array of the host. These spacers constitute immunological memories that are then used by CRISPR-associated (Cas) proteins to mount sequence-specific defence on subsequent infection. The process of acquiring new spacers is termed Adaptation and can be divided into two main stages: firstly, the generation and capture of a prespacer by Cas1, Cas2 (and potentially other) proteins and secondly, the docking of this nucleoprotein complex at the leader:repeat site, leading to integration of the new spacer by transesterification. The integration process is completed by DNA polymerase and DNA ligase. The overall process has been reviewed recently (1–3) and a schematic representation of the steps involved in adaptation in *Sulfolobus solfataricus* is shown in Figure 1A.

The first stage in adaptation is the capture of a prespacer from foreign DNA. Prespacers have no identified conserved sequences, but are found next to a short PAM (protospacer adjacent motif), which is required to guide the Cas adaptation and later interference machinery. The addition of a new spacer requires the staggered nicking of the CRISPR locus at the 5′ ends of the first repeat and the co-ordinated joining of a prespacer to the repeat ends. The joining of both ends of the spacer to the host genome occurs by two ‘half-site’ reactions, one 3′ end of the incoming DNA will be joined to the 5′ end of the first repeat, proximal to the leader sequence (site 1), and the second 3′ end of the prespacer will be joined to the leader-distal 5′ end of the first repeat on the complementary strand (site 2). Both nicking and joining occur through a one-step transesterification reaction mediated by Cas1, in which the 3′ hydroxyl residues (3′ OH) of the incoming prespacer are used to attack the host locus (4). PAM sequences are crucial for prespacer selection and integration in the correct orientation to license interference, with the prespacer end that was previously adjacent to the PAM always being inserted proximal to the leader sequence (5).

Structural studies of the *Escherichia coli* Cas1–Cas2 complex in the presence and absence of bound DNA revealed that two Cas1 dimers are joined by a central Cas2 dimer (6–8). In the DNA:Cas1–Cas2 ternary structure, tyrosine residues from two Cas1 subunits were found to bracket a 23 bp duplex and act as wedges to splay the remaining 5 bp of duplex DNA at either end into single strands. The single-stranded 3′ ends are bound tightly in an arginine-rich cleft of Cas1 and there is some evidence that they are cut five nucleotides from the end of the 23 bp duplex, at PAM sequences (5′-CTT-3′) (8). This proposed cleavage would result in the 3′ hydroxyl residues being positioned exactly in the metal-binding active site, poised to perform nucleophilic attack at the leader:repeat junction (7,8).

The insertion of new spacers is polarized and almost always occurs between leader and repeat 1 (9,10), which suggests that these elements contain important motifs that guide docking of the Cas1 and Cas2 proteins. In support of this hypothesis, the last 60 bp of the leader and the first repeat in *E. coli* were shown to be essential and sufficient for integration of new spacers (11). *Escherichia coli* Cas1 has an intrinsic sequence specificity for the nucleotides around the leader:repeat junction, suggesting that this site is targeted during the first half-site integration of a new spacer (12). However, the *E. coli* Cas1–Cas2 complex was found to integrate spacers promiscuously at the junction of each repeat in a CRISPR array and other ‘hot spots’ in pUC19 plasmid DNA, rather than uniquely at the end of repeat 1, suggesting that host factors were required for complete specificity (13). Integration host factor (IHF) was identified as the host factor, increasing specificity of integration by binding to a site in the leader sequence, bending the leader DNA and triggering recognition and docking by the adaptation complex (14,15). A similar function for IHF has recently been described for the type I-F system from *Pectobacterium atrosepticum* (16). Subsequently, the structures of *E. coli* adaptation complexes have revealed the molecular details of the integration event, highlighting the requirement for structural distortion of the target DNA, and explaining the importance of an upstream recognition motif, brought into contact with Cas1 due to IHF-mediated DNA binding, for the integration process (17).

Information on the mechanism of adaptation in organisms other than *E. coli* is more patchy, but nucleic acid sequences around the leader:repeat junction appear generally important (18–20). Recent structural studies of the *Enterococcus faecalis* type II-A adaptation process have provided a molecular framework for each stage of the integration pathway, including ternary complex formation, integration at site 1 and subsequent DNA distortion leading to full integration (21).

The work presented here focuses on the CRISPR–Cas system of *S. solfataricus*, which includes three different CRISPR–Cas types (type I-A, III-D and III-B), two different repeat families (AB and CD) and adaptation cassettes made up of genes coding for Cas1, Cas2, Csa1 and Cas4 proteins (Figure 1B). Previous studies in *S. solfataricus* have suggested that the AB and CD loci may be active for adaptation under different conditions (22). Here, we reconstitute integration *in vitro* and demonstrate that the intrinsic specificity of Cas1 is augmented by host factors in an ATP-dependent reaction. Cas1 is shown to protect prespacer DNA ends from degradation by cellular nucleases or Cas4 in a manner influenced by PAM sequences.

## MATERIALS AND METHODS

### Cloning, expression and purification

The following proteins were expressed and purified as described previously: Cas1_CD_ and Cas2_CD_ (12); Cas2_AB_ (23); Sso7 (24); Alba1 (25); SSB (26). The CRISPR DNA repeat binding protein (Cbp1) was a kind gift from Dr Xu Peng, and was expressed and purified as described (27). The Cas1_AB_ gene Sso1405 was amplified from *S. solfataricus* genome DNA by PCR using the following primer pair: (forward primer: 5′-GGCGCCATGGATAAGAAAATAGCGTTCG; reverse primer: 5′-GGTTGGATCCTCACTTCGCTAGGTATGG) and cloned into expression plasmid pEHisTev using the introduced NcoI and BamHI sites, allowing expression with a cleavable N-terminal polyhistidine tag in *E. coli* (28). The Cas1_AB_ protein was expressed and purified as described previously for Cas1_CD_ (12), with the addition of a heparin-sepharose chromatography step following removal of the polyhistidine tag. Site directed mutagenesis to generate variants of Cas1_AB_ (D234A variant), Cas1_CD_ (E142A variant) and Cas2_AB_ (D10A variant) was carried out using standard methodology and the sequences of the oligonucleotides used are available from the corresponding author on request. *Sulfolobus solfataricus* Cas4 (Sso1391) was purified as described previously (29).

### DNA substrate preparation

DNA oligonucleotides and double-stranded gBlocks were ordered from Integrated DNA Technologies (Coralville, IA, USA). If required, oligonucleotides were 5′-^32^P-radiolabelled and gel purified as described previously (16). Double-stranded prespacer substrates were formed by heating equimolar concentrations (20 μM) of complementary strands at 95°C for 5 min and then slow cooling to room temperature overnight in a heating block. The assembled substrates were purified by native polyacrylamide (12%) gel electrophoresis with 1 × Tris–borate–EDTA (TBE) buffer, followed by band excision, gel extraction, ethanol precipitation, as described previously (12). gBlocks were cloned into a pUC19 backbone according to manufacturer's instructions using EcoRI and BamHI restriction sites. All plasmid constructs were verified by sequencing (GATC Biotech, Konstanz, Germany) and gBlock sequences are available from the corresponding author on request (pCRISPR A and derivatives, pCRISPR C, pLeadArepC)

### Integration assay with radiolabelled prespacer

Cas1 and Cas2, both at 20 μM, were incubated together at 55°C for 30 min. 1 μl of this solution was then added to a reaction containing 1 μl 5′^32^P-radiolabelled DNA substrates (2 μM final) for integration (∼1% is labelled), 1 μl (100 ng/μl) plasmid DNA, 1 μl 10× integration buffer (200 mM Tris (pH 7.5), 100 mM NaCl), 1 μl MnCl_2_ (50 mM) and 5 μl water making the total reaction volume up to 10 μl. This reaction was then incubated at 55°C for 30 min. Following the incubation, 1 μl of proteinase K (20 mg/ml) (ThermoFisher Scientific) was added and the digest was incubated at 37°C for 1 h, before phenol extraction of the DNA. 10 μl of the aqueous phase containing the DNA was removed, mixed with 2 μl of 6× DNA loading dye and run on a 1% agarose gel, pre-stained with ethidium bromide, at 100 V for 1 h in 1× TBE buffer and photographed under UV light. The gel was dried for 4 h on a slab gel drier (Savant) and phosphorimaged. Plasmids were nicked with nickase Nt.BspQI (New England BioLabs) according to manufacturer instructions and run on agarose gels alongside integration assay products to act as a marker for the nicked form of the pCRISPR or pUC19 plasmids.

### PCR amplification of integration sites

A 9 μl reaction was prepared containing 200 ng of the pCRISPR A/pCRISPR C plasmids, 5 mM MnCl_2_, 1X integration buffer and 2 μM prespacer substrate (3′_overhang (see Table 1) unless otherwise stated in figure legends). 1 μl of a Cas1 and Cas2 mix (both at 20 μM) was added to this reaction and a 30 min incubation at 55°C was carried out. The reaction was phenol-extracted and the aqueous phase was diluted 1:1 with RNase-free water. 1 μl of this dilution was added to a PCR reaction containing 1μl of forward and reverse primer (IntFor and pUC19Rev1, unless otherwise stated in figure legends) (10 μM), 10 μl 2X My*Taq* Red Mix (Bioline) and 7 μl RNase-free water. The forward primer contained an *Nco*I restriction site and was complementary to the prespacer used in the integration assay. The reverse primer contained an *Xho*I restriction site and was complementary to a region of pUC19 flanking the CRISPR insert. A PCR reaction was performed consisting of an initial denaturation step at 98°C for 2 min, followed by 25 cycles of 98°C for 30 s, 55°C for 30 s and 72°C for 30 s, with a final extension for 2 min at 72°C and an infinite hold step at 4°C.

**Table 1.** DNA substrates used in this study

Double-stranded prespacer	Oligo components	Sequence (5′ to 3′)
**3′_overhang**	3′-f	TCGCCATGGTGAGCACAGAGGATAATGTAACACT
	3′-r	TACATTATCCTCTGTGCTCACCATGGCGACGAGC
**5′_overhang**	5′-f	ACACTTCGCCATGGTGAGCACAGAGGATAATGTA
	5′-r	CGAGCTACATTATCCTCTGTGCTCACCATGGCGA
**Duplex**	Dup-f	CGAGCTCGCCATGGTGAGCACAGAGGATAATGTAACACT
	Dup-r	AGTGTTACATTATCCTCTGTGCTCACCATGGCGAGCTCG
**triplePAM_prespacer**	triplePAM	TCGCCATGGTGAGCACAGAGGATAATGTACGACGACGA
	polyT	TACATTATCCTCTGTGCTCACCATGGCGATTTTTTTTT
**Primers used in SPIN assays**		**Sequence**
IntFor		TCGCCATGGTGAGCACAGAGGATA
pUC19Rev1		AATTCTCGAGTTGGCCGATTCATTAATGC
pUC19Rev2		AATTCTCGAGGGATAACCGTATTACCGCC
IntRev		TATCCTCTGTGCTCACCATGGCGA
Leader269		AATTCTCGAGGAGATAAAGAGAAAACCGG

The products of the PCR reaction were separated on a 1.5% agarose gel, which allowed rough localisation of the integration sites. PCR products selected for sequencing were cleaned up using the Wizard SV Gel and PCR Clean-Up System (Promega). Products were then digested with 1 μl NcoI and 1 μl XhoI FastDigest enzymes in a 20 μl reaction containing 1X FastDigest buffer at 37°C for 1 h. 1 μg of the pEHISTEV vector was also restricted using the same method with NcoI and XhoI to produce compatible ends for ligation of the insert. The digested inserts and plasmid were ligated and the ligation products were transformed into DH5α *E. coli* cells. Transformants were selected by overnight growth at 37°C on LB agar plates containing 35 μg/ml kanamycin. Plasmids were extracted from positive clones by Miniprep and sent for sequencing using the T7 primer (GATC Biotech). The sequences around the insertion site were used to make a sequence logo on the WebLogo server (30).

### Integration assays with *S. solfataricus* lysate

Integration assays coupled to PCR were modified by the addition of *S. solfataricus* lysate, Sso7, Alba or Cbp1 before Cas1 and Cas2 proteins. The reaction mix was set up as above without the addition of Cas proteins or RNase-free water. 1 μl of purified host proteins (from stock concentrations of 12.5–100 μM) or increasing volumes of *S. solfataricus* cell lysate (1–5 μl) (prepared as described previously (31)) were added to the reaction mix and the total volume was made up to 9 μl with RNase-free H_2_O before the addition of 2 μM Cas1 and Cas2. The reaction was completed and the products resolved as described above.

### Preparation and size exclusion chromatography of cell lysate

3.5 g of *S. solfataricus* cell pellet was resuspended in 10 ml of lysis buffer (20 mM Tris (pH 7.5), 150 mM KCl, 1 EDTA-free mini protease inhibitor tablet) and sonicated for 6 × 30 s bursts at 10 μm. The lysed cells were centrifuged at 35 000 rpm, 4°C for 30 min using the Optima L-90 K Ultracentrifuge and 70Ti rotor (Beckman Coulter). The lysate was then decanted and filtered before being used in assays. Lysate was fractionated by size exclusion chromatography and eluted in 2 ml fractions from a Superdex 200 prep grade column (GE Healthcare). Fractions were concentrated from 1.5 ml to 75 μl and 3 μl added to integration assays. Integration assays with fractionated lysate were supplemented with ATP or an ATP analogue (see figure legends for species and concentration) to retain specific integration.

### Processing of prespacer substrates

Cas1 (and where indicated Cas2) proteins (final concentration of 2 μM) were added to 20 nM prespacer substrate in a buffer containing 20 mM Tris (pH 7.5), 10 mM NaCl, 5 mM MnCl_2_ (50 mM) and 5 mM ATP. 3 μl *S. solfataricus* cell lysate or Cas4 (Sso1391) (1.5 μM) was then added and the reaction incubated at 60°C for 30 min before phenol extraction of the products and separation on a 15% denaturing polyacrylamide gel and phosphorimaging.

## RESULTS

### Reconstitution of prespacer integration by *S. solfataricus* Cas1 and Cas2

**Figure 2.**
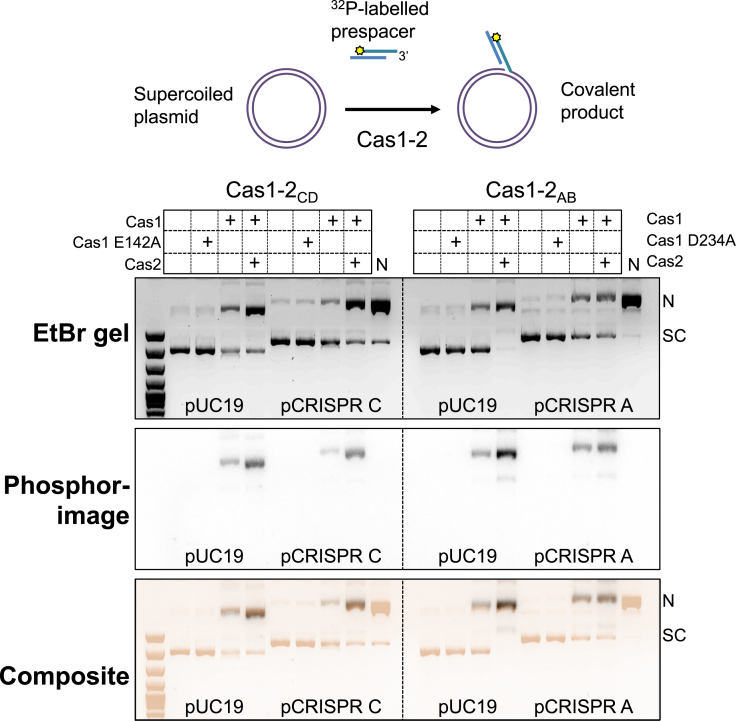
Integration of spacers into supercoiled DNA *in vitro*. Cas1–2_CD_ or Cas1–2_AB_ (2 μM) were incubated with supercoiled plasmid (100 ng) and ^32^P-labelled prespacer (3′_overhang (Table 1), 2 μM) at 55°C for 30 min in the presence of 5 mM MnCl_2_. The products of the assay were separated on a 1% agarose gel containing ethidium bromide and visualised under UV light (top). The agarose gel was then dried and phosphorimaged (middle). The scan of the pre-stained EtBr agarose and the phosphorimage of the dried gel were combined to create a composite image (bottom), with the ethidium bromide signal shown in sepia. The first lane for each substrate is a control without protein and the last lane for the pCRISPR substrates (N) is the result of nicking the plasmid with the nickase Nt.BspQI. Reactions were also set up with the Cas1 proteins alone or Cas1 active site variants, E142A Cas1_CD_ or D234A Cas1_AB._

To characterise the process of adaptation in the *S. solfataricus* type I-A system, an integration assay was developed with Cas1, Cas2 and prespacer DNA with a 5′-^32^P radioactive label. These were incubated with two supercoiled plasmid DNA species, pUC19 and pCRISPR, which is derived from pUC19 with an insert containing the CRISPR array leader, repeat and first spacer. The experiment was carried out separately with both sets of Cas1–Cas2 proteins (Cas1_AB_ and Cas2_AB_ or Cas1_CD_ and Cas2_CD_) together with the corresponding pCRISPR A or C plasmids. Wild-type Cas1 caused an increase in conversion of supercoiled (SC) to nicked (N) plasmid (Figure 2, top panel). The position of the nicked form of the plasmid corresponded with the migration of the radiolabelled prespacer, suggestive of integration (Figure 2, bottom panel). Integration was clearly enhanced by the addition of the Cas2 protein. No integration was mediated by active site variants of either Cas1_CD_ or Cas1_AB_. Both the pUC19 and the pCRISPR plasmids were good substrates for integration, suggesting that the reaction was not specific for the leader:repeat junction.

### Intrinsic specificity of Cas1 influences integration site choice

**Figure 3.**
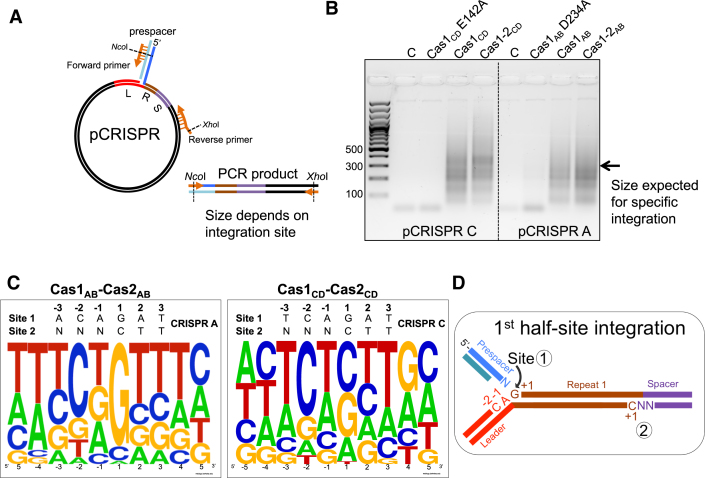
Identifying sequence motifs at Cas1–Cas2 integration sites. (**A**) Schematic of the SPIN (spacer integration) assay used to amplify integration sites. An integration reaction into the pCRISPR C or A plasmids was performed with Cas1–2_CD_ or Cas1–2_AB_ and a prespacer with 5 nt 3′ single-stranded ends (3′_overhang (Table 1)) followed by spacer-specific PCR amplification (primers IntFor and pUC19Rev1). (**B**) Analysis of PCR amplification products by agarose gel electrophoresis. The control lanes (C) show amplification from integration reactions without Cas1–2, followed by reactions with Cas1 active site mutants, Cas1 alone and Cas1–2. (**C**) A sequence logo was generated on the WebLogo server following PCR amplification, cloning and sequencing (*n* = 45) of the integration sites selected by Cas1–2_CD_ or Cas1–2_AB_. The residues are numbered as in D. The sequence of the *in vivo* integration site 1 of CRISPR A or CRISPR C is shown above the WebLogo. (**D**) Schematic of the two half-site integrations carried out by Cas1–2 during adaptation at CRISPR loci. The first half-site reaction takes place at the leader-proximal 5′ end of repeat 1 (site 1) and the second at the leader-distal 5′ end of repeat 1 (site 2). Incoming prespacer ends are shown in blue.

To assess where prespacers were being integrated into the plasmid DNA, a spacer integration (SPIN) assay was developed by coupling a standard integration reaction to PCR amplification of the integration site (Figure 3A). A forward primer complementary to one strand of the inserted prespacer with an internal NcoI site, and a reverse primer complementary to the pCRISPR plasmid with an internal XhoI site were used to amplify through the prespacer insertion sites in plasmid DNA. Integration at site 1 (the leader:repeat junction) produces a product of 323 bp for pCRISPR A and 341 bp for pCRISPR C. In the presence of active Cas1, a smear of PCR products was obtained (Figure 3B). This is consistent with integration taking place at hundreds of sites at different distances from the reverse primer, leading to the amplification of a range of products of varying sizes.

Following PCR amplification of integration sites, PCR products were digested at the primer restriction sites, ligated into the pEHISTEV plasmid (28) and clones (45 in total) sent for sequencing. Integrations were mapped all around the plasmid DNA with no apparent selection for plasmid features, such as the ampicillin resistance gene, which was found to be a hotspot for spacer insertion by the *E. coli* Cas1–Cas2 (13). The 10 nucleotides around the integration sites were also compared for sequence similarities and a sequence logo was generated using the WebLogo server (30) (Figure 3C). This revealed a clear motif present at the integration sites chosen by both Cas1–Cas2 pairs, with a preference for a C or G residue at the +1 position and for a C at the –2 position. These findings are consistent with the sequence specificity of Cas1_CD_ determined using a disintegration assay (12) and with the nucleotide sequences present at site 1 and site 2 of the *bona fide* integration site (Figure 3D). The limited intrinsic specificity of Cas1, in the presence or absence of Cas2, is clearly not sufficient to direct integration to the cognate leader:repeat site on its own, suggesting that other factors are required *in vivo*.

### Archaeal chromatin proteins do not confer specific integration

**Figure 4.**
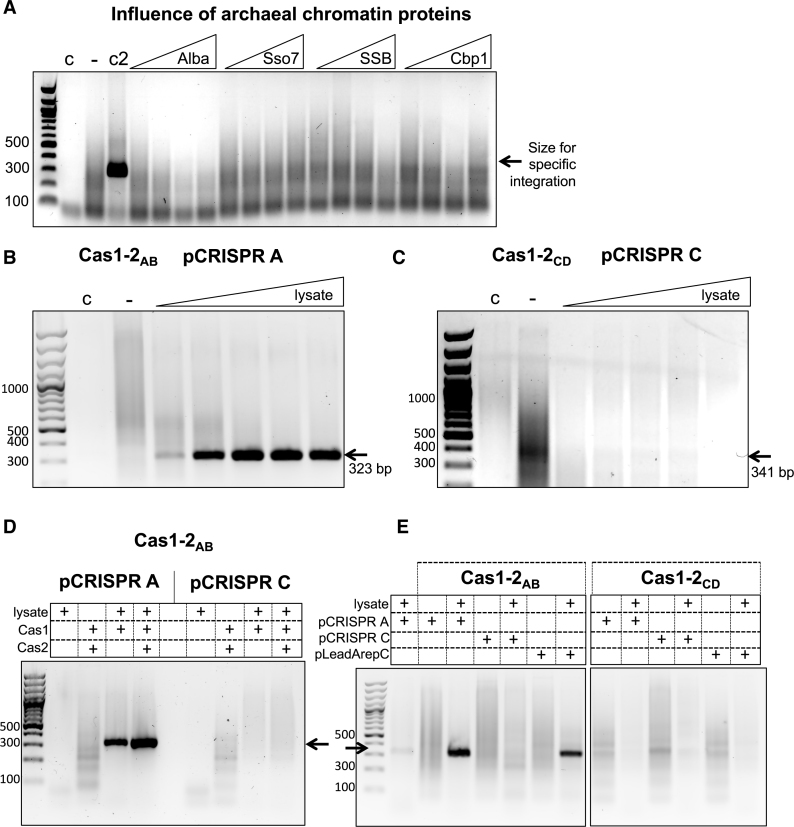
A host factor in *S. solfataricus* lysate leads to specific integration by Cas1–2_AB_. (**A**) Abundant DNA binding proteins of *S. solfataricus* were added to SPIN assays to assess whether these factors influenced integration specificity. The leftmost lane contains a 100 bp DNA ladder, followed by lanes with the amplification products from integration assays containing: cell lysate only (c); Cas1–2_AB_ (–); positive control for specific integration (c2); Cas1–2_AB_ and 1.25, 2.5, 5 or 10 μM of either Alba1 (sso0962), Sso7d (sso10610), SSB (sso2364) or Cbp1 (sso0454). (**B**) SPIN assay products following integration into the pCRISPR A plasmid by Cas1–2_AB_ with or without the addition of *S. solfataricus* cell lysate. Lanes are: DNA ladder, lysate-only control (c), Cas1–2_AB_, and Cas1–2_AB_ with 1 to 5 μl of *S. solfataricus* cell lysate added. A 323 bp product indicates correct integration at the leader-proximal 5′ end of repeat 1 (site 1). (**C**) As in B, but showing assays with Cas1–2_CD_ and pCRISPR C, where no specific products were observed. (**D**) SPIN assay with Cas1–2_AB_ and lysate, as indicated, integrating into pCRISPR A or pCRISPR C. (**E**) SPIN assay with Cas1–2_AB_ or _CD_ and lysate, as indicated, integrating into pCRISPR A, pCRISPR C or a chimeric substrate composed of the Leader from A and the Repeat from C (pLeadArepC).

Integration host factor (IHF) was shown to be important in guiding specificity of the *E. coli* Cas1–Cas2 complex to the leader-repeat junction by binding a consensus site in the leader and causing a sharp bend in this region (14,15). Given the low sequence specificity observed *in vitro* for integration by the *S. solfataricus* Cas1–Cas2 proteins, we investigated whether a similar host protein factor might be required for specific integration in this system. There is no IHF-type protein coded by *S. solfataricus*; however, the abundant DNA-binding proteins Alba1 (32) and Sso7 (33) are involved in DNA bending and compaction (34,35), and the archaeal SSB binds single-stranded DNA (36). Additionally, the protein Cbp1 (CRISPR DNA repeat-binding protein) binds specifically to the CRISPR repeats in *S. solfataricus*, opening the DNA duplex around these sites (27,37). We hypothesized that one or more of these proteins could play a role analogous to IHF in *S. solfataricus* adaptation. Accordingly, we carried out SPIN assays in the presence of increasing concentrations (0–10 μM) of these DNA-binding proteins (Figure 4A). However, no specific integration was observed in the presence of these proteins. In high concentrations of Alba1 a reduction in the smear caused by non-specific integration was observed, which may be due to this protein coating the plasmid DNA and blocking non-specific integration.

### Host factors facilitate site-specific integration of prespacers

To test the possibility that unknown host factors are required for specific integration by Cas1, SPIN assays were carried out with the addition of cleared *S. solfataricus* cell lysate to the integration reaction. As increasing volumes of lysate were added to the Cas1–2_AB_ reactions, the smear of non-specific products obtained following PCR amplification was reduced and a specific (323 bp) band appeared, consistent with integration specifically at the CRISPR A leader–repeat1 junction (Figure 4B), an observation subsequently confirmed by DNA sequencing. The absence of a specific integration product in the lysate-only condition is consistent with low expression levels of Cas1–2 in the absence of infection (38). These results confirmed that a cellular factor, or factors, guides specific integration during adaptation by Cas1–2_AB_ proteins in *S. solfataricus*. The addition of *S. solfataricus* lysate did not confer the same specificity to the integration reaction performed by Cas1–2_CD_ into the CRISPR C array, although a reduction in non-specific integration was observed (Figure 4C). Furthermore, Cas1–2_AB_ was specific for the AB locus and did not integrate prespacers specifically at the CD locus (Figure 4D). To probe this further, we designed a chimeric integration substrate by fusing the CRISPR A leader to the CRISPR C repeat (pLeadArepC). Cas1–2_AB_ integrated prespacers specifically into this chimera (Figure 4E), suggesting that the differences in the leader regions, rather than repeats, are crucial for this specificity.

### Prespacer structure influences integration

**Figure 5.**
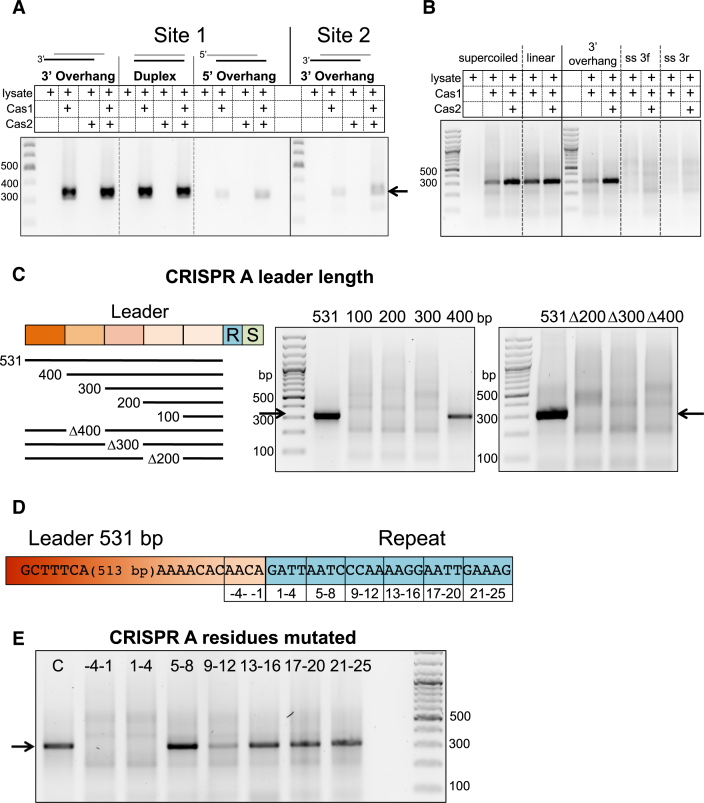
DNA requirements for specific integration. (**A**) SPIN assays carried out with prespacers with a 29 bp duplex region and either blunt ends or 3′ or 5′ single-stranded ends (5 nt) (Duplex, 3′_overhang, 5′_overhang (Table 1)), Cas1–2_AB_ and lysate, as indicated, and pCRISPR A. The first three panels show integration at site 1, the fourth shows integration at site 2 (PCR primers for site 2 amplification were IntFor and Leader269). (**B**) SPIN assays following integration of double (3′_overhang) or single-stranded prespacers (3′-f or 3′-r) by Cas1–2_AB_ into pCRISPR A in supercoiled on linearised forms, as indicated (see Table 1 for sequences). (**C**) SPIN assays following integration by Cas1–2_AB_ of prespacer DNA into the pCRISPR A leader variants in the presence of cell lysate. The first lane is a DNA ladder. (**D**) Schematic showing mutation scanning of the CRISPR A leader and repeat. (**E**) SPIN assays using pCRISPR A variants described in (D).

Prespacer end structures were varied from 5 nt single-stranded 3′ or 5′ overhangs to complete duplex ends. SPIN assays were carried out with Cas1–2_AB_ in the presence of cell lysate and primers used to amplify integrations at either site 1 (leader proximal), or site 2 (leader distal). Prespacers with 3′ single-stranded ends or blunt duplex ends were integrated efficiently at site 1 (Figure 5A). However, those with 5′ single-stranded ends resulted in very low levels of integration. The right hand panel of the image shows the products at site 2 of CRISPR A following a SPIN assay in the presence of cell lysate. A weak amplification product is present at the correct size (338 bp) in the presence of Cas1–2_AB_. We conclude that integration *in vitro* is much less robust at site 2, compared to site 1. This difference may be due to the intrinsic sequence specificity of Cas1, already described for the integration (Figure 3C) and disintegration reaction (12), leading to more efficient docking and integration at site 1, which has a better-defined sequence. Specific integration was observed at similar levels with both supercoiled and linearized plasmids, suggesting that the presence of supercoiling is not a major factor for the type I-A system. Integration was very weak with single-stranded DNA prespacers (Figure 5B), as observed recently for the type I-F system (16), consistent with the expected requirement for partially duplex DNA with two 3′ ends for full integration.

### A long leader is required for specific integration

In the well characterised type I-E system, leaders are generally less than 100 bp in length, and only 60 bp of the leader proximal to the first repeat is required for adaptation (11). In contrast *S. solfataricus*, in common with many other archaeal types, has much longer leader sequences (39). To assess the importance of the long (531 bp) CRISPR A leader sequence to integration *in vitro*, truncated versions of the leader were designed (Figure 5C) and used in SPIN assays with Cas1–2_AB_ and cell lysate. Assays with the truncated leaders showed that the minimal leader required for correct integration at site 1 *in vitro* was 400 bp, with further shortening of the leader abolishing integration at this site (Figure 5C). The amplified integration products were also much less abundant for the truncated 400 bp leader compared to the full length 531 bp substrate. Deletion of 100 bp sections internal to the leader also abolished integration. These results indicate that the full length of the long leader sequences found in systems such as *S. solfataricus* are important for specific integration, in marked contrast to the situation in types I-E, I-F and II-A.

### An intact leader-repeat junction is required for integration

To assess the importance of the repeat sequence for prespacer integration, we generated variants of the repeat-proximal leader sequence and repeat1 sequence with blocks of four nucleotides mutated (A’s were changed to C’s, T’s to G’s, and *vice versa*) (Figure 5D) for SPIN assays. When the last four nucleotides of the leader (–4 to –1) or first four nucleotides of the first repeat (1–4) were altered, integration was abolished (Figure 5E). This is perhaps unsurprising given the strong sequence selection already identified for the residues at positions –2 of the leader and +1 of the repeat imposed by Cas1 during both the disintegration and integration reaction (12). Changing the sequence of the repeat between position 9 and 12 also reduced integration at site 1. This suggests that internal motifs in the repeat are important docking sites for the adaptation complex, similar to the repeat motifs suggested to be important for adaptation complex binding and accurate repeat duplication in *H. hispanica* (19). Mutations at positions 5–8 and position 13 onwards had little effect on integration.

### Specific integration requires ATP hydrolysis

**Figure 6.**
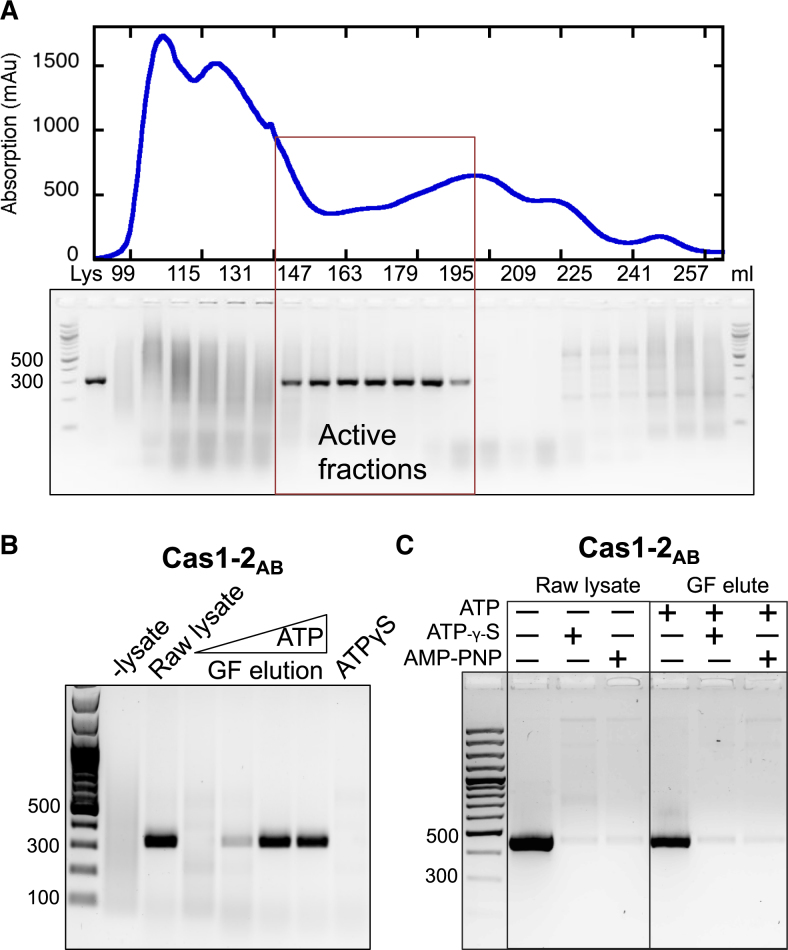
ATP is needed for host-factor mediated specific integration. (**A**) Products of a SPIN assay carried out with either raw *S. solfataricus* lysate or fractions of lysate separated by size exclusion chromatography. A trace of the peak fractions′ absorption at 280 nm is shown in the top panel. The bottom panel shows the effect addition of column fractions to an integration reaction with Cas1–2_AB_. From left to right, the lanes contain a DNA ladder, PCR from assay with raw lysate, followed by PCR from assay containing a concentrated sample of every third fraction across the elution peak. Assays with fractionated lysate were carried out in the presence of 5 mM ATP. The fractions that led to an integration product of the correct size are boxed. (**B**) Active fractions from A only facilitated specific integration by Cas1_AB_–Cas2_AB_ in the presence of hydrolysable ATP. From left to right the lanes contain a 100 bp ladder, PCR amplifications from integration assays in the presence of raw lysate, a gel filtration elution pool in presence of increasing concentrations of ATP (0, 1.25, 2.5, 5 mM). The last lane is the result of PCR amplification from the products of an assay with GF elution and the non-hydrolysable ATP analogue ATPγS (5 mM). (**C**) Comparison of integration sites chosen by Cas1–Cas2 in the presence of raw lysate or an ‘active’ pool of fractionation lysate supplemented with ATP (5 mM) and/or two non-hydrolysable ATP analogues (ATPγS and AMP-PNP) (both at 15 mM). Expected product for integration at site 1 is 449 bp here as primers IntFor and pUC19Rev2 (see Table 1) were used.

When *S. solfataricus* cell lysate was fractionated by size exclusion chromatography (Figure 6A), specific integration required the addition of ATP. Figure 6B shows the effect of adding 0–5 mM ATP to assays containing the ‘active’ fraction following separation of lysate by size exclusion. Raw lysate promoted specific integration in the absence of ATP; however, the addition of fractionated lysate in the absence of ATP resulted in no integration products of the correct size. As ATP concentration was increased a specific band at 323 bp appeared, indicating integration had taken place at site 1. The non-hydrolysable ATP analogue ATPγS did not support specific integration in SPIN assays, and addition of excess non-hydrolysable ATP analogues to raw cell lysate abolished the host-factor mediated specificity (Figure 6C). Together, these results implicate an ATP-dependent mechanism for specific integration in *S. solfataricus*. Unfortunately, the host factor could not be purified further as the activity was lost on subsequent chromatography steps.

### Prespacers are frequently processed in a PAM-specific manner during integration

**Figure 7.**
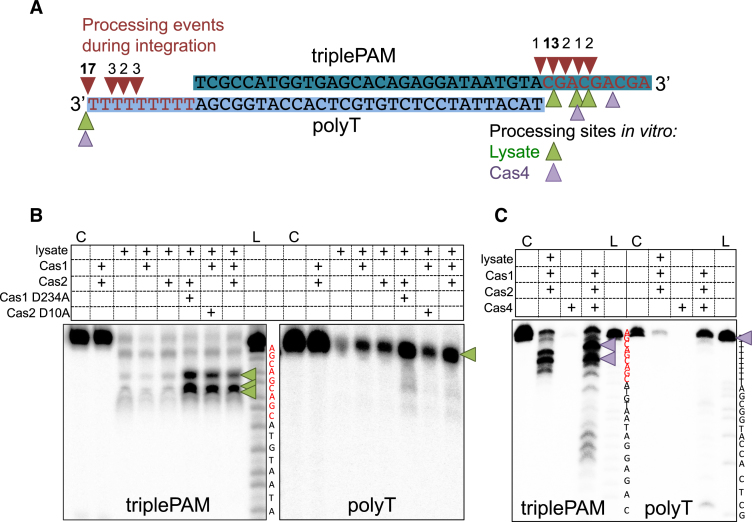
Prespacer processing is influenced by PAM sequences. (**A**) A prespacer with 3 PAM complementary sequences (5′-CGA-3′) in the 3′ single-stranded region of one strand (triplePAM) and a poly-T at the other 3′ end (polyT) (triplePAM_prespacer (Table 1) was integrated into pCRISPR A in a SPIN assay containing *S. solfataricus* cell lysate. The products of integration of either strand at site 1 were cloned and sequenced to identify processing events at the 3′ end of the prespacers. All processing events occurred in the 3′ overhangs and are indicated by red arrows, the number above indicates how many integration events were processed at each site. Green arrows indicate processing sites following incubation with Cas1–2_AB_ and cell lysate (see B), and purple arrows indicate processing in the presence of Cas1–2_AB_ and Cas4 (Sso1391) (see C). (**B**) The prespacer used in A was also incubated with Cas1–2_AB_, 5 mM MnCl_2_ and *S. solfataricus* lysate at 60°C and products run on a 15% denaturing urea–TBE polyacrylamide gel. The left hand panel shows the result of labeling the triplePAM strand and the right the result of labeling the polyT strand. The first lane is a control with only the labeled substrate loaded, followed by the products of incubation with Cas1–2_AB_ or Cas proteins in combination with *S. solfataricus* (*Sso*) lysate. Inactive mutant D234A Cas1 or D10A Cas2 were also included in incubations as indicated. An A+G ladder (L) was also loaded to map the products of triplePAM processing. Green arrows indicate the major processing products. (**C**) The same prespacer was assayed with Cas1–2_AB_ and cell lysate or Cas4 (Sso1391) to compare processing. The first lane is a control without protein (C) and an A+G ladder (L) was also loaded for each labelled species. Purple arrows indicate the major processing products.

DNA sequencing revealed that new spacers were almost invariably inserted correctly at site 1 during integration reactions containing cell lysate. Furthermore, we noticed that spacers inserted during SPIN assays in the presence of lysate often had several nucleotides removed from the 3′ single-stranded end that had been joined to the first repeat. To investigate this further a prespacer substrate was designed where one strand (triplePAM) has a 9 nt 3′ overhang containing three motifs complementary to the 5′-TCN-3′ PAM that has been identified for CRISPR loci A and B (40), whilst the complementary strand has a 9T overhang (Figure 7A). Before integration *in vivo* these PAM sequences must be removed from the prespacer end inserted at site 2 in order to license effective interference (see Figure 1A). In *E. coli* the removal of the PAM is thought to be carried out by Cas1 after a prespacer substrate is bound (8).

The sequencing analysis showed that the 3′ end containing the triplePAM was trimmed to remove at least one and frequently two or more of the three PAMs before integration (Figure 7A, red triangles). In contrast, when integrations of the complementary poly-T 3′ end were sequenced, much less processing was observed. In 17 of the 25 integrations sequenced no processing of the poly-T 3′ end had occurred and in the remaining sequences only 2-4 nts had been trimmed from the end before integration (Figure 7A).

To investigate this further, the same prespacer substrates were run on denaturing polyacrylamide gels following incubation with Cas1, Cas2 and *S. solfataricus* lysate in the same conditions used in the integration assays. Prespacers containing the triplePAM were almost completely degraded in the presence of cell lysate (Figure 7B, middle panel). However, when both Cas1 and Cas2 were present in the reaction, two products several nucleotides shorter than the full-length prespacer predominated. Both Cas1_AB_ and Cas2_AB_ together were required for the generation of these products, but their production did not require the active site of either protein, as the inactive mutants D234A Cas1_AB_ or D10A Cas2_AB_ still led to the appearance of the same processed products. In contrast, the complementary strand containing a poly-T 3′ end was not processed in the same way in the presence of Cas1–2_AB_ (Figure 7B)_._ In the presence of lysate this strand was completely degraded, and addition of Cas1–2_AB_ resulted in protection of the full-length strand, with no partly truncated products observed. These data are consistent with Cas1–2_AB_ mediated, PAM-specific processing of prespacers by cellular nucleases. This pattern of prespacer processing would result in integrated spacers with a mean size around 39 bp, in good agreement with that observed in practice (10,41)

Type I-A systems typically include a Cas4 gene as part of the adaptation module (42). The Cas4 enzyme associated with Cas1–2_AB_, encoded by *sso1391*, is a nuclease with both bi-directional exonuclease and Mn-dependent endonucleolyic activities (43) and is therefore a candidate for the nuclease activity detected in the cell lysate in these experiments. We therefore tested the effect of Cas4 (Sso1391) in our prespacer processing assays (Figure 7C). Just as for cell lysate, we observed a PAM-dependent processing of prespacers, with Cas4-mediated DNA cleavage at the PAM site (purple arrows). In marked contrast, no corresponding processing was observed in the polyT strand of the prespacer.

Together, these results indicate that the shortening of the prespacer 3′ end results from processing by a nuclease and is halted when a bound Cas1–2_AB_ complex is encountered. The presence of PAM sequences seems to direct the positioning of the Cas1–2 proteins, leading to the removal of at least one, and frequently two of the PAM sequences, while poly-T ends were fully protected by Cas1–2_AB_. The data obtained from sequencing integration sites agrees well with the processing we observed from denaturing gel electrophoresis, as the triplePAM was processed to remove PAM residues before integration, while the poly-T strand was often inserted without processing (Figure 7A).

## DISCUSSION

The CRISPR–Cas system of *S. solfataricus* is one of the most complex studied to date, with multiple CRISPR repeats and loci, adaptation modules and effector complexes (44). Here, we have focussed on the biochemistry of Adaptation, and specifically the prespacer processing and integration processes. This work has revealed a number of commonalities with other adaptation types: in particular, the requirement for key sequence motifs in the repeat and leader:repeat junction, the importance of PAMs and the preference for partially duplex prespacers with 3′-overhangs. However, in several respects adaptation in the type I-A systems appears quite fundamentally different from the well-studied I-E, I-F and II-A systems.

Firstly, there is a clear requirement for the full 531 bp length of the leader sequence for specific integration *in vitro*. Previously, a naturally occurring deletion of about 20 bp around position -50 in a CD-family leader (locus E) associated with defective adaptation in *S. solfataricus* was described (39). This CRISPR locus is very short, and new spacers have not been added since the divergence of the *S. solfataricus* P1 and P2 strains (45): observations consistent with the loss of leader sequence essential for adaptation. Extensive deletion analysis has revealed that each 100-bp section of the AB-family leader is important, with only the region beyond 400 bp non-essential. This is markedly different from the situation in I-E and II-A types, where short leaders are the norm. In type II-A systems, integration is observed with only ∼10 bp of leader sequence (21). In *E. coli* adaptation, extreme bending of the leader over a very short DNA length (∼60 bp) is accomplished by IHF binding, allowing distal regions of the leader to contact Cas1 bound at the leader:repeat junction to ensure a productive integration event (17). The abundant chromatin proteins in *S. solfataricus*, Alba1 and Sso7, do not fulfil the same function as IHF *in vitro*, as they are capable of only limited amounts of DNA bending.

Co-evolution of Cas1 with its cognate leader sequence has been observed in *S. solfataricus* and many other species (46), and clear conserved domains within the leader sequences of the *Sulfolobales* have been predicted as interaction sites for Cas protein assembly (45). Both absolute leader length and the length of core, conserved leader regions are longer in archaea than bacteria (39). Given the persistence length of DNA, which acts as a rigid rod over DNA lengths <100 bp (47), a requirement for DNA bending coupled with the lack of an IHF-like ‘super-bender’ in the archaea, could partly explain the requirement for significantly longer leader sequences. In this context, the observed requirement for ATP hydrolysis for specific integration in the type I-A system may reflect a role for energy-requiring DNA remodelling machinery in the form of helicases, DNA translocases of the SWI/SNF2 family (48) or SMC-family proteins (49). Identification of the host factors or processes important for spacer integration in the type I-A system will require further biochemical and/or genetic analysis.

The pathway of prespacer processing and capture is much less well defined than the integration process that follows. The limited evidence available on the final stages of prespacer processing points to a role for Cas1 in trimming prespacers to generate short 3′-overhangs suitable for integration. This presumed nuclease activity of Cas1 is consistent with the activity of other integrases (50)—it is essentially the same chemistry as the transesterification reaction catalysed during integration and can take place in the same active site. It also provides a neat explanation for the detection and removal of PAM sequences, but direct observation of this activity is difficult in studies linking processing to integration, as the Cas1 active site performs both roles. There is one report of PAM-directed nuclease activity by *E. coli* Cas1 *in vitro* (8), and recent studies of the type I-F system are consistent with a role for Cas1 (and not Cas3) in PAM-dependent processing of prespacer 3′ ends (16). In the type I-A system studied here, we observed no direct evidence of Cas1-mediated DNA cleavage of prespacers, regardless of the presence of PAMs. However, there was a marked PAM-dependent processing of prespacers by cellular nucleases *in vitro*, with Cas1–2 dependent trimming to remove PAM sequences. These data are consistent with PAM-dependent DNA binding of prespacers by Cas1–2 as a key step in DNA processing by nucleases. This is observed when exposing the Cas1–2:DNA complex to either cell extracts or to recombinant Cas4 exonuclease, pointing to a plausible role for Cas4 in prespacer trimming. Cas4 is essential for adaptation in the closely related organism *S. islandicus* (51) and in the type I-B from *Haloarcula hispanica* (52). It is plausible that Cas4 functions analogously to RecBCD and AddAB nucleases in these organisms (53). The sequencing data for integrated spacers fits very well with the *in vitro* nuclease data, showing that PAMs lead to extensive DNA processing whilst polyT sequences are largely untrimmed. Together, these observations are consistent with PAM-directed DNA binding of prespacers by Cas1–2 leading to protection of a spacer-sized DNA fragment adjacent to a PAM by a combination of sequence specific and ruler mediated DNA binding. The complexes may be trimmed by Cas4, potentially in combination with other host nucleases. *In vivo*, final prespacer processing could take place once the Cas1–2-prespacer complex has docked to a target DNA site, ensuring correct orientation with respect to the PAM site.

In conclusion, our study highlights the diversity in CRISPR Adaptation mechanisms across the prokaryotic domains of life. Specific integration in a type I-A system is shown to be an ATP-dependent process requiring long leaders, pointing to a possible role for active DNA remodelling. The capture and processing of prespacers, leading to integration, is one of the least understood elements of the CRISPR–Cas system. Here, we have demonstrated that the presence of a PAM sequence is a key determinant in prespacer processing, observed both from *in vitro* nuclease assays and sequencing of integration products. The nuclease activity of Cas1 is not required for this processing, but the Cas4 nuclease has been shown to possess the relevant activity, pointing to a mechanism involving PAM-directed prespacer footprinting by Cas1–2 coupled with Cas4 dependent DNA cleavage.
